# Local perceptions of cholera and anticipated vaccine acceptance in Katanga province, Democratic Republic of Congo

**DOI:** 10.1186/1471-2458-13-60

**Published:** 2013-01-22

**Authors:** Sonja Merten, Christian Schaetti, Cele Manianga, Bruno Lapika, Claire-Lise Chaignat, Raymond Hutubessy, Mitchell G Weiss

**Affiliations:** 1 Department of Epidemiology and Public Health, Swiss Tropical and Public Health Institute, PO Box, Socinstrasse 57, Basel 4002, Switzerland; 2 University of Basel, Petersplatz 1, Basel 4002, Switzerland; 3 Faculté des Sciences Sociales, Administratives et Politiques, Département d’Anthropologie, Université de Kinshasa, BP. 127, Kinshasa XI, Democratic Republic of Congo; 4 Global Task Force on Cholera Control, World Health Organization, 20, avenue Appia, 1211 Geneva 27, Switzerland; 5 Initiative for Vaccine Research, World Health Organization, 20, avenue Appia, 1211 Geneva 27, Switzerland

**Keywords:** Cholera, Vaccine acceptability, Democratic Republic of Congo, Cultural epidemiology, Mass vaccination programs

## Abstract

**Background:**

In regions where access to clean water and the provision of a sanitary infrastructure has not been sustainable, cholera continues to pose an important public health burden. Although oral cholera vaccines (OCV) are effective means to complement classical cholera control efforts, still relatively little is known about their acceptability in targeted communities. Clarification of vaccine acceptability prior to the introduction of a new vaccine provides important information for future policy and planning.

**Methods:**

In a cross-sectional study in Katanga province, Democratic Republic of Congo (DRC), local perceptions of cholera and anticipated acceptance of an OCV were investigated. A random sample of 360 unaffected adults from a rural town and a remote fishing island was interviewed in 2010. In-depth interviews with a purposive sample of key informants and focus-group discussions provided contextual information. Socio-cultural determinants of anticipated OCV acceptance were assessed with logistic regression.

**Results:**

Most respondents perceived contaminated water (63%) and food (61%) as main causes of cholera. Vaccines (28%), health education (18%) and the provision of clean water (15%) were considered the most effective measures of cholera control. Anticipated vaccine acceptance reached 97% if an OCV would be provided for free. Cholera-specific knowledge of hygiene and self-help in form of praying for healing were positively associated with anticipated OCV acceptance if costs of USD 5 were assumed. Conversely, respondents who feared negative social implications of cholera were less likely to anticipate acceptance of OCVs. These fears were especially prominent among respondents who generated their income through fishing. With an increase of assumed costs to USD 10.5, fear of financial constraints was negatively associated with anticipated vaccine acceptance as well.

**Conclusions:**

Results suggest a high motivation to use an OCV as long as it seems affordable. The needs of socially marginalized groups such as fishermen may have to be explicitly addressed when preparing for a mass vaccination campaign.

## Background

Oral cholera vaccines (OCV) are effective means to complement classical cholera control efforts in resource-constrained settings [1]; however, little is known about their acceptability in targeted communities. Community acceptance of a vaccine is the ultimate determinant of the effectiveness of any immunisation program once safe and efficacious vaccines have become available, and functional supply chains and tailored program delivery mechanisms have been established [2,3]. Clarification of vaccine acceptability prior to the introduction of a new vaccine has been stimulated by experiences with stalled immunisation campaigns in many countries [4,5].

Cholera is an acute and highly infectious diarrhoeal illness characterised by profuse watery diarrhoea and vomiting. It can lead to dehydration, shock and death within a few hours if untreated [6]. Since 2000, cholera incidence has been increasing worldwide: There are an estimated 3–5 million annual cholera cases, leading to 100,000-120,000 deaths per year [7]. Recent massive outbreaks and the failure to achieve a reduction of endemic and epidemic cholera in many regions of the world increased the awareness for a need to improve cholera control efforts. At the World Health Assembly in 2011 cholera was declared a global priority (WHA64.15), with a specific role for OCV use.

Despite many efforts, prevention through the provision of clean water and sanitation has not had the desired impact on cholera incidence [8]. As a complementary measure to improve cholera control, the use of OCVs for the prevention of larger cholera outbreaks in areas where cholera is endemic was first recommended by the World Health Organization (WHO) in 2002[9]. Two oral cholera vaccines are currently available and prequalified by the WHO [10].

The African continent accounts for most of the reported global cholera morbidity and mortality [11,12], with one of the highest concentrations of outbreaks in the Democratic Republic of Congo (DRC) [13,14]. In DRC, the first epidemics were officially reported in the 1970s, primarily in the Eastern part of the country, which has been heavily affected by two consecutive wars in the last two decades. Several areas where cholera is endemic are potentially eligible for the introduction of an OCV as a complementary means for cholera control.

### Potential barriers to vaccine acceptance

Over the last decades many well organised vaccination campaigns have been compromised by unexpectedly low participation [2,15]. In Sub-Saharan Africa three explanations have been suggested. First, information about vaccines and the disease it seeks to prevent may be insufficient at the population level, and science-based concepts of prevention may not be well understood [3,16-20]. In this situation the perceived need for prevention will be shaped by local perceptions of risk and illness [21-23]. Second, access problems linked to transport costs and time constraints on the user side, and operational shortcomings of vaccination campaigns on the health system side limit uptake [24-28]. Third, active resistance linked to political or historical conflicts may discourage use, as documented in Cameroon [29], Nigeria [19,30], and Moçambique [31,32].

This study sought to elucidate local perceptions of cholera and the potential acceptance of an OCV in a remote rural site and in a small town in Katanga province, DRC, where cholera is endemic. It also aimed to clarify the role of socio-economic and gender differentials, local illness perceptions, and to consider the social and cultural implications of illness for anticipated vaccine acceptance.

## Methods

### Study sites

The study was conducted in a rural town (Kasenga) along the Luapula River and on a fishing island (Nkolé) in Lake Mweru, Katanga Province, DRC. Kasenga has approximately 27,000 inhabitants. Electricity, water and two mobile communication networks are available. Health facilities include a district hospital, several clinics and dispensaries, and private pharmacies. Nkolé, a remote rural settlement, is inhabited by approximately 7000 people. Only one rural health centre was functional. The nearest referral centre could be reached only by boat (1 hour by speedboat, several hours by paddling).

### Study design

A mixed-methods approach combined cultural epidemiology and a qualitative rapid assessment. Cultural epidemiology integrates measurement and analysis of qualitative with complementary quantitative information from semi-structured interviews, and it aims at establishing the distribution of socio-culturally shaped illness perceptions and practices. In this study a semi-structured interview was developed and administered in a cross-sectional study to a random sample of 360 adult community respondents. It enabled us to obtain representative distributions of ideas pertinent to cholera in the absence of an outbreak, and to assess the anticipated acceptance of OCVs in the two communities.

We expected attitudes towards OCVs to be influenced by experiences during past cholera outbreaks. We therefore conducted twelve purposive in-depth interviews with affected persons and key informants who witnessed past outbreaks in order to better understand the current cholera-related local perceptions and practices. In addition, four site- and gender-separated focus group discussions were held with unaffected persons. Direct observation and informal conversation in the community (markets, restaurants, on the road) over the 3–4 weeks of data collection in each site complemented contextual information and common perceptions of sensitive issues such as witchcraft, or dissatisfaction with government healthcare providers. Data were collected by a team of four anthropologists.

### Instruments

Different instruments were used for the cross-sectional study and the in-depth interviews and focus group discussions. The cultural epidemiology approach used for the cross-sectional study required a semi-structured interview catalogue, which produced narratives as well as quantifiable answers based on the explanatory model interview catalogue (EMIC) framework of cultural epidemiology [33,34]. Since unaffected adults were interviewed, the EMIC interview began with reading a vignette that described a person with cardinal symptoms of cholera. All questions referred to this hypothetical case of cholera from the vignette. The interview incorporated questions on the socioeconomic and socio-cultural context of cholera in terms of illness-related experience (somatic and psychosocial distress), perceived causes, treatment-seeking behaviour, and previous experiences with vaccines. The selection of categories was based on prior EMIC interviews as used in Zanzibar and Kenya for the study of OCV acceptability [35,36] and on consensus among local experts. Questions further addressed active demand for a vaccine and anticipated acceptance of an OCV. Different price levels were assumed to assess the priority assigned to a vaccine in view of competing needs (free vaccine; cost of USD 1; USD 5; USD 10.5). The maximum price corresponded with the Dukoral purchase price at the time of planning the study.

The EMIC interview follows a specific interviewing and coding procedure. Open questions are asked first, and answers are coded using a list of predefined categories. Subsequently, respondents were asked to identify the most important category among all the categories they had mentioned. This scoring facilitated analysis of the variation in cultural illness-related beliefs and practices, and of anticipated oral cholera vaccine acceptance and its determinants. Questions addressing anticipated stigma were assessed using Likert scales. The vignette and questions were translated into two local languages, i.e. ciBemba and Kiswahili.

Quantitative analysis of the EMIC interview proceeds with a deductive approach, as it requires the a priori definition of the possible answer categories. Narratives to the open EMIC questions complement the quantitative data, indicating various socio-cultural constructs. Although it enables the researcher to relate narratives to quantitative codes for specific respondents, the instrument is more limited for in-depth discussions of an emerging theme that would allow an inductive approach. We therefore conducted additional in-depth interviews to further contextualise the quantitative findings in their local background. Open questions addressed past experiences with cholera outbreaks. In this way links between the prominence of socio-cultural response categories and the framework of underlying social processes and economic forces could be addressed. Interviews were conducted and recorded in the local languages.

### Sampling strategy, data collection and informed consent

Data were collected in August and September 2010 by locally recruited interviewers fluent in two local languages; they were trained for 10 days prior to the fieldwork. For the cross-sectional study, the random walk method was used to select households. Based on census data it was estimated that in Mwalimu, Kasenga, every 21st and in the village of Nkolé every 7th household had to be visited. Starting from a main place a random number between 1 and 10 was selected to identify the first household. Interviews were conducted with the head of the household or the spouse. Adults above 18 years of age who had lived in the residence for at least six months were included. If no eligible respondent was found, the next household was selected. For the interviews with key informants health professionals and local authorities were contacted and asked for consent to be interviewed. Health professionals were then asked to identify and contact persons with personal experience of cholera for an in-depth interview. Participants for focus groups were contacted in the same way and comprised non-affected persons who had however been living in the community during past outbreaks. Every participant signed an informed consent form prior to their interview. Ethical approval was obtained from the University of Kinshasa.

### Data management

Interviews were recorded in original language. Narratives were then transcribed and translated into French by the interviewers using f4 V. 4.0. Coding for thematic content was done with MAXQDA 10. Categorical data from EMIC interviews was double entered in EpiInfo V. 3.5.1 and converted for statistical analysis in SAS V. 9.2.

### Approach to analysis

The analysis was conducted in two stages triangulating different epistemological and methodological dispositions. Statistical analysis of the EMIC data provided distributions of illness-related experience, meaning, help-seeking behaviour, and attitudes towards prevention. It also enabled assessment of determinants of anticipated OCV acceptance. While this first part of analysis takes a deterministic stance and aims at answering testable questions, in a second part, narratives were coded and interpreted in order to better understand the local meaning of cholera and its prevention.

First the quantitative analysis is presented. Frequencies and prominence values (importance assigned to an item) of response categories for questions about the perceived causes of cholera, its prevention, treatment seeking, and the potential social impact of cholera, are presented graphically.

Anticipated OCV acceptance at different assumed cost levels (free, USD 1, USD 5, USD 10.5) was calculated. To examine socio-cultural determinants of anticipated acceptance of OCVs, categories answering the above questions about the perceived causes of cholera, its prevention, treatment seeking, and the potential social impact of cholera, are considered as potential explanatory variables. For response categories related to the same underlying concept, indices were calculated. The following items were selected for inclusion in a cumulative index: a hygiene index combined ‘hand washing’, ‘clean water’, ‘clean/safe food’, ‘safe garbage disposal’, ‘safe disposal of stool’, and ‘health education’. A social impact index combined ‘fears of being isolated’; ‘fears to infect others/blame’; and ‘interference of cholera with social relationships’ while items, which related to felt or internalized stigma such as ‘feeling shame’ were assessed separately. Cronbach Alpha was used to test internal reliability of the indices and sum scores were calculated.

Due to the near-universal anticipated acceptance of an OCV at no cost and a low variation at a low cost of USD 1 logistic regression could only be conducted for an assumed expenditure of USD 5 (medium) and USD 10.5 (high). For both outcomes (anticipated OCV acceptance in case of costs of USD 5, and anticipated OCV acceptance in case of costs of USD 10.5) univariable logistic regressions were conducted with SAS V. 9.2 for the following factors: cholera-related experiences (somatic and psychosocial distress and stigma), perceived causes of cholera, treatment-seeking behaviour, previous experiences with vaccines, and sociodemographic factors including sex, age, education and main source of income. Each response category was tested for interaction with sex and with site. Following the univariable analysis, every response category that related to the same question was included in a separate intermediate model (not shown) if either the p value for being associated with the dependant variable was <0.2, or if the p value for interaction with sex or site was <0.1. Intermediate models were additionally controlled for socio-economic covariates. From the intermediate models all determinants with p values <0.2 for main effects or p values <0.1 for interactions were included in a comprehensive multivariable model. Variables were retained in the comprehensive model if their p value was below 0.2.

While the comprehensive model allowed assessing the effect of each answer category and did not include the index variables, we were also interested in assessing a cumulative effect of the social impact and hygiene variables. Therefore the multivariable analysis was repeated, replacing the hygiene and social impact categories with the respective index. Based on the same criteria as for the individual answer categories a comprehensive model containing the indices (index models) was created for each outcome (acceptability at USD 5 and USD 10.5).

### Approach to qualitative data analysis

Narratives from the open questions of the EMIC interview for the 360 respondents, the four focus group discussions and twelve in-depth interviews were transcribed and imported into MAXQDA. Narratives of the EMIC were initially grouped with reference to a specific question eliciting the narrative. The narratives were read several times and coded for thematic content. The codes were then grouped into larger thematic areas pertinent to cholera and vaccine acceptability to achieve theoretical saturation. Findings were then compared with the results from the quantitative analysis to elaborate and triangulate with reference to the framework of underlying social processes.

## Results

### Study population

With the EMIC interview 181 women and 179 men from the two sites were interviewed. Two women refused to participate (participation rate 99%). In-depth interviews were conducted with three men and three women in each site (n = 12) enquiring about personal experiences with cholera. In each of the four focus group discussions, ten persons that were not affected by cholera participated (20 men, 20 women).

From the randomly sampled EMIC respondents of the town of Kasenga, half of the participants were peasant farmers while others pursued independent income-generating activities (Table 1). In contrast, on the more remote island of Nkolé, fishing was the largest source of income (30.5%). Overall, 12.5% were formally employed and 21.7% reported no personal income, the majority being women from the fishing island. The average monthly income of a household was USD 48 (median USD 15).

**Table 1:** Characteristics of study population

	**Rural town (N = 180)**	**Fishing village (N = 180)**	
**Respondents’ characteristics**			
Mean (median) age	39.7(38)	37.2(35)	(*)
Married (%)	78.3%	83.9%	
No education (%)	5.0%	7.8%	
University (%)	6.7%	1.7%	*
**Household composition**			
Mean (median) household size	6.1(6)	6.3(6)	
Mean (median) number of children	3.3(3)	3.2(3)	*
**Monthly household income**			
Mean (median) household income in $	49(15)	47(15)	
Reliable income (%)	29.4%	41.1%	*
**Main source of income**			
Agriculture (%)	50.0	11.1	***
Fishing (%)	2.2	30.6	***
Self-employment (%)	16.1	21.7	
Formal employment (%)	9.4	8.3	
Housewife	10.6	25.6	***

(*) p < 0.1 * p < 0.05 *** p < 0.001.

#### Recognising cholera

After having listened to a short vignette about a person affected by profuse watery diarrhoea, 96% of EMIC respondents identified the affliction as cholera. Experiences with cholera were generally widespread within the community: nearly every second respondent (44%) reported that a family member had experienced cholera, and 8% of the people had been affected themselves. All but one respondent considered cholera as potentially fatal without timely and appropriate treatment.

#### Perceived causes of cholera, and treatment seeking practices

**Figure 1:**
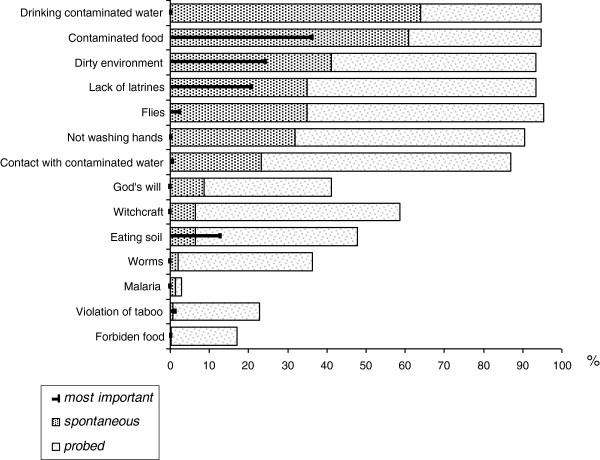
Perceived causes of cholera (%).

The majority of EMIC respondents considered insufficient hygiene and sanitation levels as the key cause of cholera. Ingestion of contaminated water or food was spontaneously mentioned by 63% and 61% as main sources of cholera. Other common explanations were contact with contaminated water, or flies, a dirty environment, lack of latrines and not washing hands (Figure 1). In contrast magico-religious explanations were rarely mentioned spontaneously (<10%). However, after probing 59% of respondents confirmed sorcery as possible source of cholera. Similarly, eating soil, and God’s will were confirmed by 48% and 41% of respondents as possible origins of cholera after probing. The importance of witchcraft was reflected in the narratives with reference to past outbreaks. In a key informant interview a nurse from Kasenga pointed out that witchcraft beliefs used to lead to delayed treatment seeking: ”Sometime back it was bad. The fact that people who were walking simply dropped and were in a coma made people think directly of witchcraft. The first thing they did, they went to the traditional healer. Only if this didn’t work, then the people were taken to the clinic. That’s why there were many deaths…. Only when people realized that witchdoctors died as well they started to doubt that it was witchcraft”.

**Figure 2:**
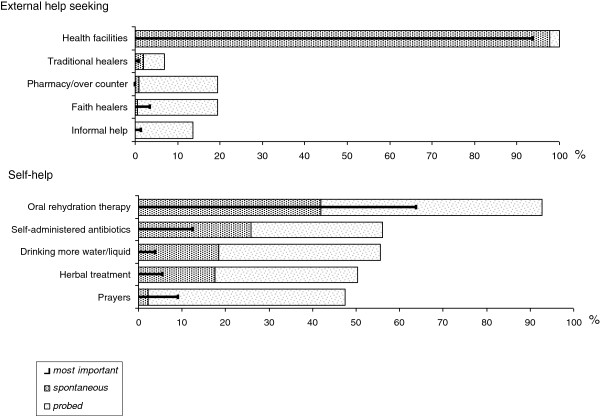
Treatment seeking (%).

Meanwhile many people had experienced several cholera outbreaks. When respondents were asked about the adequate treatment of cholera, most (99%) said they would go to a health facility, despite the continuing presence of witchcraft beliefs. Oral rehydration therapy was the most common self-help treatment that was mentioned spontaneously (42%). Other treatment practices, such as traditional medicine or religious practices were far less often mentioned (Figure 2).

#### Perceived psychosocial and material implications of cholera, and effect on everyday life

**Figure 3:**
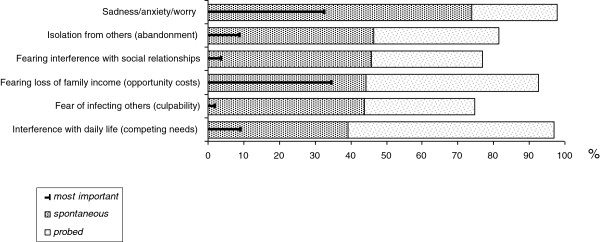
Psycho-social impact of cholera.

Respondents were further asked about the psychosocial distress caused by cholera. Over three-quarters of EMIC respondents spontaneously mentioned anxiety (74%), fear of isolation (46%), or adverse effects on social relationships (46%) (Figure 3). Fishermen and their spouses were more likely to mention the negative social consequences (p < 0.001). The precarious social consequences were substantiated in the narratives of affected persons as in the case of a fisherman from Kasenga who reported that ”all friends left because they were scared of being infected”. Given the severity of cholera, affected persons depended on the help of others for care and treatment, which was a common explanation of concerns about social support in the narratives of EMIC respondents as well.

Financial constraints due to cholera were another common concern, mentioned spontaneously by 44% of EMIC respondents (Figure 3). Material constraints further restricted the willingness to assist a sick person, in addition to their fear of being infected: In another key informant interview a fisherman professed that he hesitated to take his sick colleague to the clinic because he feared that he would have been expected to pay for his treatment.

The common fears of the social and material implications of cholera were accompanied by a certain degree of anticipated stigma against affected persons. Questions that asked specifically for stigma and discrimination of cholera patients revealed that 48% of respondents confirmed at least one of the following statements: A person with cholera would hide the disease (15%), or be ashamed (18%), experienced problems with neighbours or others (18%) or would be denied help from the family (20%). In the narratives stigma was based on the assumption of improper behaviour of those affected by cholera, extending from personal hygiene to sexual mores: “People will think bad about [the sick person] saying she might even have HIV, so she will be avoided”, as a female EMIC respondent from Kasenga suggested. Another respondent thought that “having cholera will lower [the sick person] in society because he is a dirty person since he has got this disease of dirty hands”. (EMIC interviews, male respondent, Kasenga). In addition, being affected by cholera may invoke accusations of causing harm to others. In the words of another female EMIC respondent who commented on the vignette story “[the sick person] is scared to infect others as they would then know who infected them”.

#### Community perceptions of cholera prevention

**Figure 4:**
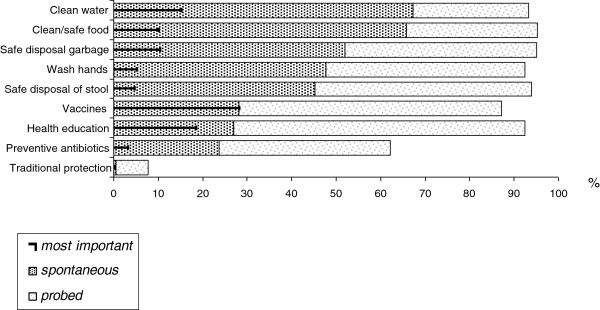
Prevention of cholera (%).

When asked about ways to prevent cholera, most respondents mentioned spontaneously the availability of clean water, and food hygiene (67% and 66%), mirroring the most common perceived causes. Vaccines were however considered the most *efficient* prevention measure: 28% of respondents gave priority to vaccines, followed by health education (18%) and the provision of clean water (15%) (Figure 4).

**Figure 5:**
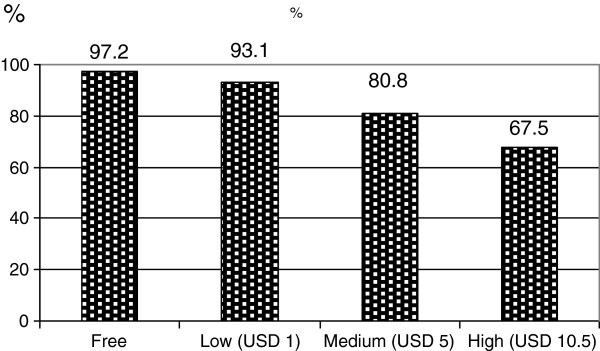
Anticipated cholera vaccine acceptance (N = 360) at different price levels (%).

Although less than a third of the respondents gave priority to a vaccine for cholera prevention, anticipated acceptance of an OCV if it would be available for free was nearly universal: 97% of participants confirmed that they would be willing to use an OCV at no cost (Figure 5). Anticipated acceptance declined when assumed cost increased: 93% said they would take an OCV if it costs USD 1, 81% if it costs USD 5, and 67.5% if it costs USD 10.5. No differences between sites and according to the sex of the respondent could be observed. However, fishermen and their spouses were significantly less likely to anticipate OCV acceptance at the highest price (P = 0.020).

#### Determinants of anticipated acceptance of OCVs

In the second part of this paper we examine to what extent the local perceptions of cholera, which are described above, influenced anticipated OCV acceptance (Tables 2–3). In addition, socio-economic determinants are investigated.

**Table 2:** Multivariable logistic regression assessing social and cultural determinants of anticipated OCV acceptance at medium price in Katanga, DRC, N = 360

**Anticipated vaccine acceptance at medium price (USD 5)**							
	**Comprehensive model**				**Index model**		
	**OR**	**95% CI**	**P**		**OR**	**95% CI**	**P**
**Perceived causes**				**Perceived causes**			
Witchcraft	- ^a^			Witchcraft	1.40	(0.95-2.07)	0.093
**Prevention**				**Prevention**			
Safe disposal of faeces - Women	2.51	(1.33-4.73)	**0.004**	INDEX ^b^ hygiene knowledge	2.81	(1.45-5.45)	**0.002**
Safe disposal of faeces - Men	1.19	(0.74-1.92)	0.466				
No prevention known	0.48	(0.25-0.92)	**0.027**				
**Faith-based practices**				**Faith-based practices**			
Prayers - Women	0.78	(0.58-1.06)	0.112	Prayers - Women	0.75	(0.56-1.01)	0.061
Prayers - Men	4.88	(2.00-11.86)	**<0.001**	Prayers - Men	3.86	(1.63-9.16)	**0.002**
**Social impact**				**Social impact**			
Interference with social relationships	0.63	(0.47-0.83)	**0.001**	INDEX ^c^ social impact	0.46	(0.26-0.82)	**0.008**
**Stigma**				**Stigma**			
Would not disclose cholera	0.42	(0.19-0.90)	**0.026**	Would not disclose cholera	0.46	(0.21-0.98)	**0.046**
**Socio-demographic variables**				**Socio-demographic variables**			
Household size (+1)	0.92	(0.85-1.01)	0.068	Household size (+1)	0.93	(0.86-1.01)	0.093
< 2 years of school	0.13	(0.05-0.37)	**<0.001**	< 2 years of school	0.15	(0.06-0.42)	**0.002**

^a^ Not retained in final model because p > 0.2. Other variables that were not retained in the multivariable model but were significant in the univariable analysis were age, employment, hand washing as prevention. Other Sociodemographic indicators tested for inclusion but were not significant were educational level, income, fishing, marital status, site.

^b^ Replaces individual items and combines ‘hand washing’, ‘clean water’, ‘clean/safe food’, ‘safe garbage disposal’, ‘safe disposal of stool’.

^c^ Replaces individual items and combines ‘fears of being isolated’, ‘fears to infect others/blame’, and ‘interference with social relationships’.

**Table 3:** Multivariable logistic regression assessing social and cultural determinants of anticipated OCV acceptance at high price (USD 10.5) in Katanga, DRC, N = 360

**Anticipated vaccine acceptance at high price (USD 10.5)**							
	**Comprehensive model**				**Index model**		
	**OR**	**95% CI**	**P**		**OR**	**95% CI**	**P**
**Perceived cause of cholera**				**Perceived cause of cholera**			
Unprotected/spoiled food	1.32	(1.07-1.64)	**0.011**	Unprotected/spoiled food	1.30	(1.05-1.61)	**0.018**
God’s will	0.73	(0.55-0.98)	**0.034**	God’s will	- ^a^		
**Cholera prevention**				**Cholera prevention**			
Safe disposal of garbage	1.26	(0.99-1.61)	0.065	INDEX ^b^ hygiene knowledge	2.91	(1.55-5.47)	**<0.001**
**Faith-based practices**				**Faith-based practices**			
Prayers - Women	1.20	(0.90-1.59)	0.211	Prayers - Women	1.13	(0.85-1.49)	0.403
Prayers - Men	1.82	(1.15-2.86)	**0.010**	Prayers - Men	1.86	(1.16-2.98)	**0.010**
**Social impact of cholera**				**Social impact of cholera**			
Fear of infecting others - Women	0.80	(0.56-1.16)	0.239	INDEX ^c^ social impact	0.42	(0.26-0.69)	**<0.001**
Fear of infecting others - Men	0.55	(0.37-0.81)	**0.003**				
**Stigma**				**Stigma**			
Would not disclose cholera	- ^a^.			Would not disclose cholera	0.55	(0.28-1.10)	0.091
**Financial impact of cholera**				**Financial impact of cholera**			
Loss of family income	0.85	(0.73-0.98)	**0.026**	Loss of family income	0.84	(0.72-0.98)	**0.025**
**Sociodemographic variables**				**Sociodemographic variables**			
Household size (+ 1)	0.94	(0.87-1.01)	0.092	Household size (+ 1)	0.93	(0.87-1.00)	0.062

^a^ Not retained in final model because p > 0.2. Other variables that were not retained in the multivariable model but were significant in the univariable analysis were age, employment, fishing, interference with daily life, anxiety, loss of income in case of cholera. Other Sociodemographic indicators tested for inclusion but not significant in the univariable analysis were educational level, income, marital status, site.

^b^ Replaces individual items and combines ‘hand washing’, ‘clean water’, ‘clean/safe food’, ‘safe garbage disposal’, ‘safe disposal of stool’, and ‘health education’.

^c^ Replaces individual items and combines ‘fears of being isolated’, ‘fears to infect others/blame’, and ‘interference with social relationships’.

#### Socio-economic variables

Having gone to school for less than two years was significantly associated with reduced OCV acceptability if it would cost USD 5 to take the vaccine (Table 2). At a higher price of USD 10.5 education showed no effect. Instead, persons who stressed the financial implications of cholera were less likely to anticipate OCV acceptance (Table 3). Income as such was not directly associated with anticipated acceptance, and also the effect of living in a larger household, where resources might be diluted, was only marginally significant.

#### Effect of perceived causes and prevention of cholera on anticipated OCV acceptance

Cholera-specific knowledge positively influenced OCV acceptability at an assumed price of USD 10.5: Respondents who considered the ingestion of contaminated food as a cause of cholera were more likely to anticipate OCV acceptance. Similarly, women who recognised the importance of safe disposal of faeces to prevent cholera were more likely to anticipate OCV acceptance (Table 2, comprehensive model). We wanted to know if overall knowledge of hygiene influenced OCV acceptability for both sexes. Instead of individual answer categories we assessed the effect of a sum-score combining ‘hand washing’, ‘clean water’, ‘clean/safe food’, ‘safe garbage disposal’, and ‘safe disposal of stool’. We found a strong and consistent association of the score with OCV acceptability irrespective of the assumed price (Tables 2–3, index model). While knowledge of hygiene-related measures to prevent cholera was positively associated with anticipated vaccine acceptance, prioritizing a vaccine for prevention was not. Prior experiences with vaccines (own or in family) showed no effect on anticipated acceptance either.

While correct knowledge of cholera was positively associated with anticipated OCV acceptance, respondents who believed that cholera was due to the will of God were less likely to anticipate acceptance (Table 3, comprehensive model). This finding was challenged by the fact that men who reported praying for healing were *more* likely to anticipate OCV acceptance. Apart from religious beliefs and practices, the local belief systems, including witchcraft beliefs, were not showing any significant association.

#### Effect of psychosocial factors on anticipated OCV acceptance

Anticipating an adverse social impact of cholera was negatively associated with OCV acceptability: Respondents who feared that cholera would interfere with social relationships were significantly less likely to accept a vaccine at a cost of USD 5 (Table 2, comprehensive model). In addition respondents who believed that a cholera infection is better not disclosed to others were less likely to anticipate OCV acceptance at a cost of USD 5. The effect was not significant in the USD 10.5 model (Tables 2–3). At a higher cost the fear to (be held responsible for) infecting others was negatively associated with anticipated OCV acceptance (Table 3, comprehensive model), even though only for men.

To assess the cumulative impact of several social implications on vaccine acceptability, a model including a sum score combining ‘fears of being isolated’; ‘fears to infect others/blame’; and ‘interference of cholera with social relationships’ was analysed. The score of social implications was associated with a lower anticipated acceptance of OCVs irrespective of the assumed price of the vaccine (Tables 2–3, index model). Unwillingness to disclose cholera was included separately in the model. It was negatively associated with anticipated OCV acceptance in the USD 5 model as well.

## Discussion

Past experiences with cholera outbreaks created high awareness and fear of cholera in this remote area in South-Eastern DRC. Health education and the provision of clean water have curbed cholera incidence to some extent, but resources were lacking to maintain protected wells and boreholes and diarrhoeal illness continues to impinge on the population. This is the background to the high acceptability of an oral cholera vaccine in this cholera-endemic area. The high willingness to pay may somewhat overestimate respondents’ actual ability to pay (hypothetical bias) [37]. Cost-related barriers have been repeatedly reported to negatively affect vaccine acceptance [16,24,27,38-40]. That costs may play a role in the study sites as well is supported by the fact that respondents who considered the loss of income in case of cholera a problem were *less* likely to anticipate OCV acceptance if a higher cost would have to be met. Efforts to reach the most vulnerable people may therefore be required if equity in access is to be achieved. Factors linked to a vaccination campaign itself, such as opening hours, could not be assessed in absence of a campaign but may affect uptake as well, as a similar study conducted in Zanzibar showed [41,42].

Our results equally showed that respondents who feared the social impact of cholera, such as interference with social relationships, were less likely to anticipate OCV acceptance This may seem counter-intuitive at first sight because we would expect a person who fears the consequences of cholera to be more interested in prevention. But the fear of losing social support because of cholera needs to be understood in this context of broader social insecurity. In African settings access to resources is usually mediated by group membership, mainly the family, or clan. Where formal social security is lacking, group membership is often the only way for the poor to mobilise material support during a crisis such as illness [43]. Investments in social networks are important coping strategies [44]. The lower anticipated OCV acceptance of respondents who stress the social implications of cholera may reflect their material insecurity and weak social networks rather than their reluctance to use a vaccine. Prior research has come to similar conclusions. Cassell et al. (2006) reported from Gambia that mothers with a weak social network were less able to access childhood vaccination [20].

In our study fishermen and their families were more likely to anticipate social and financial implications of cholera. In the unadjusted analysis they were also less likely to anticipate acceptance of an OCV at the highest cost, even if their monthly income was not lower as compared to others. Fishermen often live under difficult conditions. Already in the 1990s van Bergen had described a ‘poverty-complex’ of poor living conditions, frequent migration, disrupted social structures, and problems around alcohol consumption and sexual transactions providing a breeding ground for both HIV and cholera in the fishing camps of lake Mweru [45]. An agency report from 2009 confirms that for example poverty-related fish-for-sex exchange in Kasenga and the surrounding fishing camps are locally perceived a problem and a cause of HIV in the area [46]. HIV and poverty have been described to be a problem in fishing villages in other settings in the region as well [47-49]. The lower acceptability of a vaccine at a high cost among fishermen in the unadjusted analysis is therefore likely to be explained through greater social and economic vulnerability in the multivariable models. The identification of vulnerable subgroups such as the fishermen and fish traders may help to improve equity in access to a vaccination campaign, because there is often a predisposition to neglect difficult-to-reach people out of logistic reasons especially if herd protection may already be achieved by a relatively low coverage as was shown to be the case for cholera [50].

A process of social marginalization can be reinforced if a feared disease is thought to be the problem of a particular group of people. To explain the effects of both negative impact and concern about stigma of cholera on anticipated vaccine acceptance, one may consider reluctance to accept a vaccine as a kind of anticipatory coping with dreaded social exclusion and stigma by denying vulnerability to cholera. Negative attitudes of health professionals towards poor and marginalized people may additionally compromise acceptance of a vaccine. In other contexts a patronising and disrespectful treatment by health professionals has been shown to discourage especially poor and marginalised persons from using (childhood) vaccination services [20,27].

On the individual level, education and information about vaccination are known to influence vaccine acceptance [51,52]. In our study education influenced OCV acceptability at a lower price as well, while at a higher price material insecurity became more important. But cholera-related knowledge influenced vaccine acceptability irrespective of the price. In Zanzibar, where a similar study was conducted, individual-level barriers to OCV acceptance during a mass vaccination campaign included unawareness of the infectious pathways and symptoms of cholera as well [42]. It was notable that local illness beliefs, like witchcraft or the breach of a taboo, were not associated with OCV acceptability in DRC. Other research in Ghana found as well that traditional practices had no influence on the readiness to use vaccines [20]. Hence local traditional health beliefs and practices do not necessarily compete with science-based approaches. Nonetheless there is a possibility of underreporting of traditional beliefs and practices in our study, and delayed treatment-seeking because of witchcraft beliefs might still be a problem even if the acceptability of a vaccine is not compromised.

Besides traditional beliefs, Christian religious practices were common, without being mutually exclusive. In contrast to traditional beliefs, faith-based practices – namely praying for healing and the belief that God was responsible for cholera outbreaks – did show an effect on anticipated OCV acceptance, however in a contradictory way. The literature mentions an ambiguous influence of religion on health-related behaviour as well. Religiosity has been associated with a higher sense of control over one’s health [53]. Conversely, prevention such as vaccinations may be perceived as interfering with God’s plans [32]. Similarly ambiguous were the findings in this study. Prayers for healing, which are positively associated with OCV acceptability, may on the one hand indicate more active coping with health problems at the individual level and reflect active church membership and a strong social support network. On the other hand, respondents who mentioned God’s will to be at the origin of cholera were less likely to anticipate OCV acceptance. Persons who lack agency and autonomy in particular have been shown to ascribe to fatalistic positions [54]. They may be less optimistic about their participation in a vaccination campaign, which too, may play a role for vaccine acceptability.

### Limitations of the study

Household selection took place at random, and only two persons per site refused to participate in the study. Nonetheless, a potential underreporting or misreporting of local practices for treatment and prevention due to the negative connotation of these practices within the health system cannot be excluded (desirability bias). But witchcraft beliefs for example, which were common in the area, were not negatively associated with anticipated acceptance, suggesting that traditional medical beliefs and practices are not necessarily a barrier to vaccine acceptability. Nonetheless these results should be interpreted with caution. Witchcraft narratives were often linked to local authorities and politicians, suggesting limited trust in government or other authority. As the cross-sectional study did not include questions related to quality of care or trust in healthcare providers this could not be further investigated.

## Conclusions

The town of Kasenga and the fishing island of Nkolé are prototypical sites in DRC where cholera has remained endemic, despite attempts to improve the sanitary infrastructure. Respondents demonstrated active interest in a vaccine and anticipated OCV acceptance was nearly universal. Nonetheless, there are several risks. In this setting, social insecurity and costs linked to vaccination may jeopardise the use of OCVs especially for socially marginalized individuals and groups, and social mobilization is likely to be key for the success of any vaccination campaign. Collaboration with local authorities who know the dynamics in their communities is therefore important. Furthermore, in order to better meet the expressed needs of the population for an improved water and sanitation infrastructure, any vaccination campaign should be combined with other cholera control activities, such as water and sanitation rehabilitation and the provision of health education.

## Competing interests

The authors declare that they have no competing interests.

## Authors’ contribution

CS, MGW and SM conceived of the study, with the support of BL, CM, CC, and RH. SM and CM conducted the study and analyzed the data, with the support of CS and MGW. SM drafted the manuscript. All authors commented on earlier drafts and read and approved the final manuscript.

## Authors’ information

CC and RH are staff members of the World Health Organization. The authors alone are responsible for the views expressed in this publication and they do not necessarily represent the decisions, policy or views of the World Health Organization.

## Pre-publication history

The pre-publication history for this paper can be accessed here:

http://www.biomedcentral.com/1471-2458/13/60/prepub
